# Effects on musculoskeletal pain, work ability and sickness absence in a 1-year randomised controlled trial among cleaners

**DOI:** 10.1186/1471-2458-11-840

**Published:** 2011-11-01

**Authors:** Marie B Jørgensen, Anne Faber, Jørgen V Hansen, Andreas Holtermann, Karen Søgaard

**Affiliations:** 1 National Research Centre for the Working Environment, Lersø Parkallé 105, 2100 Copenhagen, Denmark; 2 Institute of Sports Science and Clinical Biomechanics, University of Southern Denmark, Odense, Denmark

## Abstract

**Background:**

Only a few workplace initiatives among cleaners have been reported, even though they constitute a job group in great need of health promotion. The purpose of this trial was to evaluate the effect of either physical coordination training or cognitive behavioural training on musculoskeletal pain, work ability and sickness absence among cleaners.

**Methods:**

A cluster-randomised controlled trial was conducted among 294 female cleaners allocated to either physical coordination training (PCT), cognitive behavioural training (CBTr) or a reference group (REF). Questionnaires about musculoskeletal pain and work ability were completed at baseline and after one year's intervention. Sickness absence data were obtained from the managers' records. Analyses were performed according to the intention-to-treat-principle (ITT).

**Results:**

No overall reduction in musculoskeletal pain, work ability or sickness absence from either PCT or CBTr compared with REF was found in conservative ITT analyses. However, explorative analyses revealed a treatment effect for musculoskeletal pain of the PCT. People with chronic neck/shoulder pain at baseline were more frequently non-chronic at follow-up after PCT compared with REF (p = 0.05).

**Conclusions:**

The PCT intervention appeared effective for reducing chronic neck/shoulder pain among the female cleaners. It is recommended that future interventions among similar high-risk job groups focus on the implementation aspects of the interventions to maximise outcomes more distal from the intervention such as work ability and sickness absence.

**Trial registration:**

ISRCTN: ISRCTN96241850

## Background

High physical work demands increase the risk of musculoskeletal pain [1,2], impaired work ability [3] and long-term sickness absence [4,5]. Moreover, musculoskeletal pain, work ability and high sickness absence are predictors of early retirement from work [3,6,7]. Thus, the consequences of musculoskeletal pain, impaired work ability and elevated sickness absence are considerable for the individual as well as for society [8,9].

There are indications that low educated job groups which are often characterised by high physical work demands, high sickness absence and early retirement have less access to health initiatives through the workplace compared with their more highly educated counterparts [10]. Therefore, effective well-documented initiatives to prevent musculoskeletal pain, impaired work ability and sickness absence among workers with high physical work demands are needed [11].

It is well documented that female cleaners have high physical work demands [12]. Data from a representative sample of the Danish workforce reveal that cleaners have an elevated amount of work involving pushing and pulling tasks and twisted postures with bent neck and twisted back or squatting, and cleaners characterise their job as physically strenuous [13]. Thus, although cleaning work is not heavy as in construction work or repetitive as in assembly line work, the cleaning tasks involve day-long raised physical exposure. Therefore, cleaning work is properly characterised as a job with high physical work demands. Cleaners also report high levels of musculoskeletal pain in almost all regions of the body, but especially in the neck/shoulder region and the lower back [12,14]. Furthermore, they have an elevated risk of early retirement [6] and have recently been reported as having poor to moderate work ability [14]. Although many physically heavy jobs have been taken over by machines, cleaning is a permanent job segment in all countries, with on-going challenges in coping with the physical demands of the work [2]. Thus, initiatives to reduce the occurrence or recurrence and the consequences of musculoskeletal pain are needed in this job group. For the cleaners' work and symptom profile, such initiatives should be both preventive and therapeutic, addressing the interaction between musculoskeletal pain and daily physical exposure.

A number of ergonomic and ergonomic organisational adaptations have been applied to cleaning work and some of these have been evaluated. Unfortunately, they have seldom proved effective [15]. This may be caused by these adaptations often being followed by increasing work pace and cut backs, which ultimately will increase work demands again. While the final solution to these problems remains to be found, a new, promising method for relative exposure reduction emerged in the literature. Previously, physical training has been shown to be efficient in reducing musculoskeletal pain among patients [16] and within occupational settings among workers with sedentary repetitive jobs [17-20]. In addition, one study on office workers has shown the preventive potential of physical training on musculoskeletal pain [21]. Physical training has also been found to improve work ability among sick-listed workers [22] and a few studies have found positive effects from physical training on sickness absence [23-25]. However, among workers with high physical work demands, no randomised controlled interventions with physical training have reported beneficial effects on musculoskeletal pain, work ability or sickness absence [26,27]. Because physical training is known to increase physical capacity [28], and thereby to decrease relative physical workload [2], physical training should in theory be particularly effective in preventing and reducing musculoskeletal pain, impaired work ability and sickness absence among workers with high physical demands.

Cognitive behavioural therapy (CBT) has been shown to be efficient in reducing days with sickness absence in a return-to-work programme [29], and musculoskeletal pain in primary care [30]. Furthermore, CBT has been shown to improve measures of coping such as catastrophising [31] and pain-related fear of physical activity. Pain-related fear of physical activity has proven to be disabling and thus influential for work ability and sickness absence [32,33]. Since both pain and physical movement are highly evident among cleaners, CBT initiatives towards reducing pain-related fear of physical activity may improve work ability and reduce sickness absence. However, the effects of CBT interventions for primary prevention of musculoskeletal pain, reduced work ability and sickness absence among working employees at high risk have not been previously investigated.

In summary, several trials on treatment efficiency of physical training and CBT have proved successful among patients [20,34]. However, such efficacy trials are typically tested in optimal implementation contexts among highly motivated patients in a clinical setting [35,36], and the results are not necessarily transferable to workplace interventions among non-patients [36]. To establish the effectiveness of physical training and CBT in primary and secondary prevention, trials should be conducted in workplace settings and high-risk workers are a relevant group to target. However, high quality intervention effectiveness trials in workplaces with physically demanding work are few, primarily unsuccessful and lacking in the literature [2,37].

We have previously analysed the results of the first three months of the interventions, and found that physical coordination training improved abdominal muscle strength and postural balance, and that cognitive behavioural training improved fear of movement related to pain (kinesiophobia) [38]. The current paper focuses on the long-term effects of the intervention - 12 months after baseline. Accordingly, the aim of this trial was to test the effectiveness of this workplace intervention consisting of physical coordination training or cognitive behavioural training on musculoskeletal pain (secondary outcome), work ability and sickness absence (primary outcomes) among female cleaners.

## Methods

The study is a part of the previously described FINALE programme [2] aimed at investigating preventive initiatives against physical deterioration (musculoskeletal pain, poor work ability and sickness absence) among work groups with high physical work demands. The study was a cluster randomised controlled intervention conducted at nine cleaning workplaces in Denmark. Approval was received from the Scientific Ethics Committee of the Capital Community, Denmark (H-C-2007-0033) and the randomised controlled trial was registered with a unique trial registration number (ISRCTN96241850).

### Recruitment procedure

Participants were recruited from cleaning workplaces in the urban, rural and metropolitan regions of Zealand, Denmark. Hospitals, cleaning companies, and large businesses with in-house cleaning services situated in the target area, were identified through internet search, union and company networks, or from common knowledge in the research department. In order to be able to randomise participants into clusters, an inclusion criterion for a workplace was that at least 30 cleaning employees were required to be engaged in the same geographical area. Furthermore, workplaces needed to be able to offer the intervention either as part of the employees' working day or give the employees an opportunity to be compensated with overtime when it was spent participating in the interventions. Participant inclusion criteria were that they had to be employed for at least 20 hours/week at the workplace and primarily work during day hours. Their main work task had to be cleaning, but their job could also involve other service tasks such as washing, kitchen work or attending to patients. No exclusion criteria applied to participation in the intervention study. All eligible employees were invited to an information meeting during working hours and asked to complete a screening questionnaire on whether to enrol or not to enrol in the study (further information on the data from the screening questionnaire content has been reported previously) [39]. For employees who did not attend the information meeting, managers subsequently handed them written information on the project and screening questionnaires with a stamped addressed envelope. Those who consented to participate were invited to answer a questionnaire. Further details of the recruitment procedure have previously been reported [39].

### Randomisation procedure

For the cluster randomisation procedure, each workplace was considered a stratum. Clusters depended on work teams where possible or were made up of either groups where employees had lunch together, or groups where they worked in close proximity to each other, or groups who reported to the same manager. Clusters were matched on sex, age and job seniority. The randomisation was performed with the help of blinded staff casting a lot. Unfortunately, blinding of participants was not possible in such a workplace intervention study.

### Design

The cleaners were randomised into either physical coordination training, or cognitive behavioural training or a reference group. Both data collection and interventions were conducted during the cleaners' working hours. Questionnaires were collected and physical tests conducted at baseline and at 12-month follow-up.

### Interventions

The interventions were delivered in two phases: an intensive phase of 3 months' duration followed by a less intensive phase of 9 months' duration.

#### Physical coordination training

In the first intensive intervention phase, weekly 20-minute sessions were performed at the workplace with guidance from an instructor. The physical coordination training consisted of intensive physical coordination exercises providing high activation of stabilising muscles around the trunk and shoulder girdle and was evaluated in a pilot study [40]. This intensive training programme was designed to improve muscular strength and postural stability of the cleaners [40]. In the second phase comprising the following 9 months, the number of training sessions was gradually reduced, with only one session per month during the last 6 months. In this phase, the participants were introduced to several new types of physical exercise training based on the participants' preferences.

#### Cognitive behavioural training

The cognitive behavioural training occurred in groups guided by a trained group leader, and was also divided into two phases. The first intensive intervention phase consisted of a 2-hour session at the work place twice a month. The cognitive behavioural training mainly comprised group discussions of issues regarding pain-related dysfunctional attitudes like kinesiophobia, coping and management, with facilitation of functional alternatives. Moreover, the training involved education in physical activity, problem-solving, applied relaxation techniques and practice of the coping skills in the home environment [41]. In the second phase, the number of training sessions was gradually reduced, with only one session of 1 hour's duration per month during the last 6 months. In this phase, the experiences and considerations of the cognitive and behavioural changes of the participants from the first phase were debated, and reflections on and support for obtaining long-lasting cognitive and behavioural changes were the focus.

#### Reference group

The reference group received a health check of 1 hour's duration, including a pulmonary-function test and an aerobic capacity test [42]. The intention of the health check was to give the participants some attention and encourage their participation in the test and questionnaire components.

### Outcome measures

#### Musculoskeletal pain

A structured self-administered questionnaire on musculoskeletal pain was distributed with a modified version of the Standardised Nordic questionnaire for the analysis of musculoskeletal symptoms [43]. The following question was posed: "How many days have you had trouble in [body part] during the last 12 months?" (0 days, 1-7 days, 8-30 days, 30-90 days, more than 90 days, every day). The question was posed with [body part] replaced by the neck, right shoulder, left shoulder and low back. Since this study involved all workers at the workplace, several of the participants did not have musculoskeletal pain (i.e. neck pain < 30 days last year n = 64%), and had no possibility to improve their musculoskeletal pain level. Therefore, an explorative analysis including only participants with musculoskeletal pain at baseline was performed. In this manner, the potential of the physical coordination training to decrease the musculoskeletal pain level was examined. Moreover, to investigate the potential of the cognitive behavioural training to prevent musculoskeletal pain, an additional exploratory analysis including only participants without musculoskeletal pain at baseline was carried out. For this purpose the answer categories were dichotomised into non-chronic (0-30 days) and chronic (> 30 days) musculoskeletal pain. Similar case definitions have been used previously [44], and > 30 days of musculoskeletal pain has been found to be consistent with clinical diagnosis of chronic musculoskeletal pain [45].

#### Work ability

Work ability was evaluated with a question asking self-reported current work ability compared with their self-assessed lifetime best work ability on a scale from 0-10. This question is one item in the validated work ability index (WAI) [46], shown to have a similar validity as the whole index [47] and to be sensitive to changes over time [48].

#### Sickness absence

Sickness absence data were retrieved from local databases maintained by the employers at the nine workplaces. The records listed the beginning and the end dates of each sickness absence for each employee 6 months before the intervention started until the end of the intervention. The number of sickness spells and days for each month were registered. In Denmark, employees are paid their full salary during sick leave, and the employer is compensated for longer sickness absence spells (spells longer than 15 days up until June 1^st ^2008 and spells longer than 21 days after June 2^nd ^2008) by the state health insurance system. To receive this compensation, the workplaces are required to keep precise records of sickness absence. The number of days with sickness absence represents workdays only. Maternity leave and absences attributable to caring for a child are not included in the sickness absence registrations. Absence due to caring for a child is allowable for up to two consecutive days with no upper limit in days per year. Similarly, reasons for sickness absence such as medical diagnoses were also not registered.

### Data and statistics

To perform a conservative intention-to-treat (ITT) analysis, the following two-step procedure was applied. To account for drop-outs, missing data were imputed from the last available data observation. That is, if a participant dropped out before the 12-month follow-up, baseline data were carried forward based on a conservative assumption that the intervention effect equalled zero among those dropping out. In this way, conservative estimates of the intervention effects were obtained by applying an appropriate (specified for each outcome below) statistical model to observed and imputed data (step one). Then, since the amount of imputed observations could likely underestimate the standard errors of intervention effects, standard errors were computed by applying the same statistical model on the observed data only (step 2). The significance of the effects was then assessed by comparing effect estimates from the first step with standard errors from the second step.

Analysis of the between randomised groups differences in drop-out was performed with a general linear model with completers and baseline characteristics as random effects for the numeric variables (age, BMI and work ability) and with a Pearson's chi squared test for the categorical variables (neck, right shoulder, left shoulder and lower back pain).

#### Musculoskeletal pain

The effects of interventions on the dichotomised version of musculoskeletal pain in each of the four body parts: neck, right shoulder, left shoulder and low back, were evaluated following the ITT principle, using logistic mixed effect models with person-identification and workplace included as random effects to account for the multi-level design of the study.

Further, analyses on the preventive and treatment potential for musculoskeletal pain were made [21]. The data regarding musculoskeletal pain were dichotomised into two different categories: chronic (> 30 days the last year) or non-chronic (0-30 days the last year) at baseline and follow-up, respectively. Change in symptom status in terms of the proportions of participants who changed to non-chronic at follow-up given a chronic condition at baseline and vice versa was investigated. The participants were defined as having musculoskeletal pain in the neck/shoulder if they had trouble in at least one of the three body parts (neck, left shoulder, right shoulder). This analysis was performed on the ITT data as well, with observations carried forwards and backwards in the case of missing observations at follow-up or baseline, respectively. P-values for positive effect of interventions were obtained by Fisher's exact test in one-sided analyses.

#### Work ability

The effects of interventions on work ability were estimated to be analogous to the estimation of effects on musculoskeletal pain, applying a Gaussian mixed effect model.

#### Sickness absence

The number of spells of sickness absence in the 6 months prior to baseline and follow-up respectively was modelled using a mixed effect Poisson model, analogous to the analyses on musculoskeletal pain and work ability. The effect of interventions on the number of sickness absence days in the 6 months prior to follow-up was modelled using the Mann-Whitney U test. The same test was applied to absence days prior to baseline to test similarity of intervention groups at baseline. Sickness absence analyses were based on the ITT principle.

## Results

**Figure 1:**
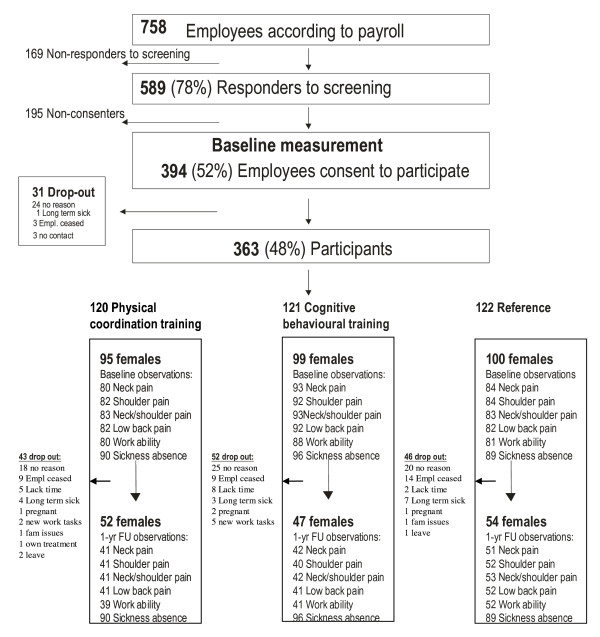
**Flow of participants during the study indicating recruitment, randomisation and drop-out**. Yr = year, FU = follow-up, Empl. = employment.

From nine workplaces, 758 eligible employees were invited to participate in the study. A screening questionnaire was distributed at an information meeting at each workplace and 589 responded as to whether they consented to participate. There were 394 cleaners who consented to participate and after baseline testing, 363 participants were enrolled in the randomisation (Physical coordination training n = 120, cognitive behavioural training n = 121, reference group n = 122). Few males (n = 69) were part of this study. Potential gender differences in all the outcomes exist. While the inclusion of males in the analyses would have increased overall power, the resultant addition of this skewed gender covariate would have reduced the statistical power available to detect intervention effects. Therefore, analyses are based on female cleaners only. The flow of participants is illustrated in Figure 1. Mean participation rate in the intensive intervention phase was 29% for physical coordination training and 48% for cognitive behavioural training.

The baseline characteristics of the participants for each of the three intervention groups are given in Table 1. No systematic bias seems to have been introduced from the randomisation procedure. A comparison between the baseline characteristics among completers and drop-outs in the three randomisation groups is given in Table 2. There were no statistically significant differences between the randomised groups in the characteristics of those dropping out. However, numerically there seem to have been more without pain dropping out of the intervention groups (physical coordination training and cognitive behavioural training) than in the reference group.

**Table 1:** Baseline characteristics of the participants in each of the three intervention groups.

	Physical coordination training		Cognitive behavioural training		Reference group	
	(N = 95)		(N = 99)		(N = 100)	
	Mean	SD	Mean	SD	Mean	SD
Age (yrs)	44	9.1	46	8.9	45	9.6
Height (cm)	160	7.1	161	7.7	163	7.8
Weight (kg)	73	14.5	72	17.1	73	14.5
BMI (kg/m2)	28	5.1	28	5.9	28	5
Job seniority (years)	9.4	9.1	9.9	8.1	10.3	9.6

N = number of participants, SD = standard deviation

**Table 2:** Baseline characteristics among those cleaners completing the study (completers) and those dropping out of the study (drop-outs)

	**Physical coordination training**		**Cognitive behavioural training**		**Reference group**	
	**Completers**	**Drop-outs**	**Completers**	**Drop-outs**	**Completers**	**Drop-outs**
**Percent (n)**	**55 (52)**	**45 (43)**	**47 (47)**	**52 (52)**	**54 (54)**	**46 (46)**
	
Age years (SD)	46 (8.9)	43 (9.2)	47 (8.1)	44 (9.3)	46 (8.9)	43 (10.1)
BMI kg/m2 (SD)	28.5 (4.9)	27.9 (5.5)	28.2 (6.0)	27.3 (5.9)	27.6 (4.5)	27.5 (5.7)
Neck pain (% > 30 days)	59.5	44.4	51.4	32.5	37.5	37.9
Right shoulder pain (% > 30 days)	50.0	21.9	48.6	30.8	30.6	36.0
Left shoulder pain (% > 30 days)	35.9	25.8	33.3	30.8	23.3	51.7
Lower back pain (% > 30 days)	58.8	38.5	42.4	39.3	42.1	54.2
Work ability 0-10 mean (SD)	7.5 (1.8)	7.7 (2.3)	7.5 (1.9)	7.5 (2.3)	7.6 (1.9)	7.1 (2.4)

n = number of participants, SD = standard deviation

### Musculoskeletal pain

**Figure 2:**
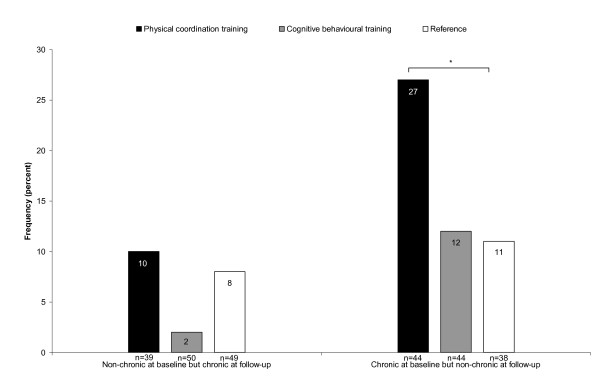
**Change in pain status (neck/shoulder)**. Frequency of participants who changed their pain status in the neck/shoulders (i.e. from chronic pain at baseline to non-chronic pain at follow-up and vice versa) in the three intervention groups. * = significant difference between intervention groups (physical coordination training or cognitive behavioural training) and reference group at p < 0.05 in Fisher's exact test, n = number of participants.

**Figure 3:**
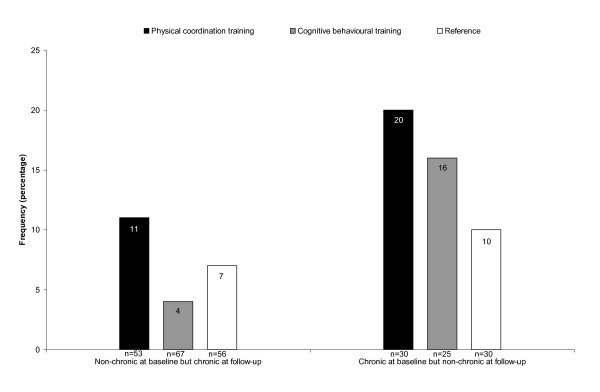
**Change in pain status (low back)**. Frequency of participants who changed their pain status in the low back (i.e. from chronic pain at baseline to non-chronic pain at follow-up and vice versa) in the three intervention groups. There was no significant difference between intervention groups (physical coordination training or cognitive behavioural training) and reference group from Fisher's exact test, n = number of participants.

There was no significantly different reduction in musculoskeletal pain in the neck, shoulders or low back after the interventions compared with the reference group (p > 0.05). The baseline and follow-up levels are shown in Table 3. However, explorative analyses of prevention and treatment potential for musculoskeletal pain (i.e. persons changing from a chronic to non-chronic condition, and vice versa) revealed effects of the physical coordination training intervention compared with the reference group in an ITT analysis, as shown in Figures 2 and 3.

**Table 3:** Primary outcomes from baseline to follow-up in intention-to-treat sample.

		Physical coordination training			Cognitive behavioural training			Reference		
		
		n	Baseline	Follow-up	n	Baseline	Follow-up	n	Baseline	Follow-up
Neck pain	(%> 30 days)	82	41	37	93	33	30	86	30	27
Right shoulder pain	(%> 30 days)	83	33	27	91	33	29	87	25	25
Left shoulder pain	(%> 30 days)	82	27	23	90	30	29	86	27	28
Lower back pain	(%> 30 days)	83	36	36	92	27	26	86	35	36
Work ability	(mean 0-10 (SD))	81	7.6 (2.0)	7.8 (1.9)	89	7.5 (2.1)	7.5 (2.1)	87	7.3 (2.2)	7.4 (2.4)
Sickness absence days	(Median)	95	4.0	4.25	99	3.0	3.0	100	3.0	2.0
Sickness absence	(Mean spells)	95	1.622	1.356	99	1.281	1.354	100	1.64	1.281

n = number of observations (including observations carried forwards and backwards for intention-to-treat analyses), SD = standard deviation, Spells = number of sickness absence periods.

### Work ability

There was no difference in the change of work ability from baseline to follow-up between the groups in the ITT analysis (p > 0.05). The baseline and follow-up levels are shown in Table 3.

### Sickness absence

There was no significant difference in accumulated sickness absence between the groups at either baseline (the 6 months before the trial) or at follow-up (the last 6 months of the trial) (Table 3). There was also no significant difference in change of number of sickness absence spells from baseline to follow-up between groups (Table 3).

## Discussion

The main finding of this workplace intervention study among female cleaners is the reduced number of female cleaners with chronic musculoskeletal pain as a result of the physical coordination training. First, this is the first time a reduction in chronic musculoskeletal pain in a randomised controlled workplace trial among this high-risk job group of female cleaners has been demonstrated. Second, the effect was relatively large with 27% turning from chronic to non-chronic in the physical training group compared with 11% in the reference group. Third, the effect is shown even in an ITT analysis. Therefore, the risk of bias due to drop-out or level of adherence rate is eliminated. Furthermore, the relief in chronic neck/shoulder pain is likely to have a considerable influence on the daily function and well-being of this group which has high physical demands involving repeated arm and shoulder movements [12,49]. The finding in this study among female cleaners with high physical demands supports the positive results on musculoskeletal pain of physical strengthening and coordination training in previous studies among workers with low physical demands [16,17,21].

No effects on work ability from the interventions were found in the current study. Work ability is influenced by several individual characteristics and work-related factors [50]. Therefore, the lack of effect on work ability may be because the improvements in musculoskeletal pain, strength, balance and kinesiophobia found as a result of the current interventions were too small and thus didn't impose sufficient impact on the work ability. This explanation is supported by the low adherence and high drop-out rates in the current study, which ultimately water down the effect in the ITT analysis. A previous study on a similar job group with weekly physical exercise sessions for 8 months with high adherence and low drop-out found modest improvements in work ability [51]. Therefore, more frequent training sessions and a multi-disciplinary approach may be necessary for improving work ability in high-risk job groups.

No effects on sickness absence from the interventions were found. This finding is in accordance with other studies [21,51]. This may be that the effect on the mediating factor for sickness absence reduction (musculoskeletal pain) was insufficient for reducing sickness absence. Moreover, the sickness absence data were very unevenly distributed, which hindered the use of parametric repeated measures analyses. Furthermore, our measure of sickness absence was not related to a specific disease or condition. Thus, non-specific sickness absence is difficult to change with interventions aimed at musculoskeletal pain only. Perhaps effects could be found on a more specific sickness absence measure, for example sickness absence related to musculoskeletal pain.

### Strengths and limitations

The interventions of this study were performed in a standardised design following the CONSORT statement with blinded randomisation, control group, and analysed on a conservative ITT basis. Such a design reduces the risk of bias due to drop-out or the effects from other initiatives in the workplace during the intervention. It also allows for an interpretation of the effectiveness of applying physical coordination training and cognitive behavioural training in a workplace setting.

However, power limitations may have existed in this study. First, the recruitment for the study was not optimal [39] for attaining the intended power. Originally, our power analysis assumed that there would be a drop-out rate of 20%. With 125 individuals in each intervention group, this would result in a final number of 100 individuals per group to be included in the analyses. The power calculation was based on the variation of work ability, and showed that 10% changes would be assessable with a significance level of 0.05 and a power of 0.9. Second, the study suffered from the low adherence rate and high drop-out rate resulting in a lower dose of the interventions than may be needed for attaining significant effects. The negative results and the low adherence rates may be explained by either the innovation of the interventions, or the implementation of the interventions. First, the interventions were novel and adapted to a workplace setting through pilot studies and have proven efficacious in previous trials. However, further modifications may be necessary in order to fit the interventions to a workplace setting with inclusion of both high- and low-risk individuals. For example, the interventions seem to have generated less interest among those with lower pain at baseline, than among those with higher pain at baseline according to the drop-out analyses presented in Table 2. Second, the implementation of the interventions was not fully obtained, as is shown by the relatively low adherence rates. Possible barriers to the implementation may be related to the characteristics of the organisational context. For example, low influence on work tasks and poor financial circumstances are evident among cleaners [52]. Recently, barriers related to lack of personnel resources have been reported in workplace interventions [53]. Furthermore, a number of unanticipated events occurred during the intervention (to be reported in a separate paper), which highly correlated with the adherence rate. In short, higher numbers of unanticipated events (i.e. events related to the management, the organisation of the work, the production and the delivery of the intervention (shifting instructors)) were associated with lower adherence rates. Thus, the interventions seem to have been sensitive to the context of cleaning workplaces and the feasibility of the intervention should probably be reconsidered for future interventions in similar workplaces.

Another suggested reason for the low adherence could be that this RCT intervention was not implemented at the organisational level of the workplace, and this can be a barrier for good implementation [54]. Moreover, almost half of the drop-out was related to ceased employment, new work tasks, pregnancy, leave or long-term sickness absence, all of which are inevitable events, especially in the cleaning sector.

The authors suggest that future workplace interventions involve the whole organisation and existing work environment structures in the intervention process. A means to combine this with a scientific approach could be to use the stepped wedge design, to involve all workers in a department in the same intervention [55]. Interventions to maintain work ability and reduce musculoskeletal pain and sickness absence among cleaners should probably address prevention from multiple dimensions by: (i) combining both physical coordination training and cognitive behavioural training with organisational level and (ii) participatory ergonomics to obtain provable effects on the outcomes. Finally, thorough process evaluations should be conducted to point out barriers to implementation and issues that may modify the relation between the intervention and the outcome.

## Conclusions

The physical coordination training intervention seems effective for reducing chronic neck/shoulder pain among the female cleaners. Cognitive behavioural training showed indications of a prevention effect on neck/shoulder pain, but this non-significant indication should be interpreted with care. Due to low adherence and high drop-out rates, no firm conclusions can be drawn with respect to the effects of the interventions on work ability and sickness absence. It is recommended that future health-promoting intervention studies among similar job groups focus on the implementation aspects of interventions at both the individual and organisational level.

## Competing interests

The authors declare that they have no competing interests.

## Authors' contributions

MBJ, KS, Overall design. MBJ, KS, Design of the PCT-intervention. MBJ, KS, AFH, Design of the CBTr-intervention. AFH, Conduct of the CBTr-intervention. MBJ, KS, JVH, AHO, Design of data analyses. JVH, Statistician, conduct of data analyses. MBJ, First drafting of the manuscript. All authors read and approved the final manuscript.

## Pre-publication history

The pre-publication history for this paper can be accessed here:

http://www.biomedcentral.com/1471-2458/11/840/prepub
